# MDM2 SNP309, gene-gene interaction, and tumor susceptibility: an updated meta-analysis

**DOI:** 10.1186/1471-2407-11-208

**Published:** 2011-05-29

**Authors:** Yan Wan, Wei Wu, Zhihua Yin, Peng Guan, Baosen Zhou

**Affiliations:** 1 Department of Epidemiology, School of Public Health, China Medical University, Shenyang 110001, China; 2 Key Laboratory of Cancer Etiology and Intervention, University of Liaoning Province, China; 3 China Medical University Center For Evidence-based Medicine, Shenyang 110001, China

## Abstract

**Background:**

The tumor suppressor gene *p53 *is involved in multiple cellular pathways including apoptosis, transcriptional control, and cell cycle regulation. In the last decade it has been demonstrated that the single nucleotide polymorphism (SNP) at codon 72 of the *p53 *gene is associated with the risk for development of various neoplasms. *MDM2 *SNP309 is a single nucleotide T to G polymorphism located in the *MDM2 *gene promoter. From the time that this well-characterized functional polymorphism was identified, a variety of case-control studies have been published that investigate the possible association between *MDM2 *SNP309 and cancer risk. However, the results of the published studies, as well as the subsequent meta-analyses, remain contradictory.

**Methods:**

To investigate whether currently published epidemiological studies can clarify the potential interaction between *MDM2 *SNP309 and the functional genetic variant in *p53 *codon72 (Arg72Pro) and *p53 *mutation status, we performed a meta-analysis of the risk estimate on 27,813 cases with various tumor types and 30,295 controls.

**Results:**

The data we reviewed indicated that variant homozygote 309GG and heterozygote 309TG were associated with a significant increased risk of all tumor types (homozygote comparison: odds ratio (OR) = 1.25, 95% confidence interval (CI) = 1.13-1.37; heterozygote comparison: OR = 1.10, 95% CI = 1.03-1.17). We also found that the combination of GG and Pro/Pro, TG and Pro/Pro, GG and Arg/Arg significantly increased the risk of cancer (OR = 3.38, 95% CI = 1.77-6.47; OR = 1.88, 95% CI = 1.26-2.81; OR = 1.96, 95% CI = 1.01-3.78, respectively). In a stratified analysis by tumor location, we also found a significant increased risk in brain, liver, stomach and uterus cancer (OR = 1.47, 95% CI = 1.06-2.03; OR = 2.24, 95%CI = 1.57-3.18; OR = 1.54, 95%CI = 1.04-2.29; OR = 1.34, 95%CI = 1.07-1.29, respectively). However, no association was seen between *MDM2 *SNP309 and tumor susceptibility in the stratified analysis by *p53 *mutation status (GG vs TT: OR = 1.17, 95% CI = 0.75-1.82 and TG vs TT: OR = 1.09, 95% CI = 0.89-1.34 for positive *p53 *mutation status; GG vs TT: OR = 0.95, 95% CI = 0.72-1.25 and TG vs TT: OR = 1.06, 95% CI = 0.85-1.30 for negative *p53 *mutation status).

**Conclusions:**

The analyses indicate that *MDM2 *SNP309 serves as a tumor susceptibility marker, and that there is an association between *MDM2 *SNP309 and *p53 *Arg72Pro regarding tumor susceptibility. Further studies that take into consideration environmental stresses and functional genetic variants in the *p53*-*MDM2*-related genes are warranted.

## Background

The p53 protein is a principal mediator of growth arrest, apoptosis, and senescence in response to an array of cellular damage [1-3]. Various types of stress can induce high levels of p53 protein, thus preventing inappropriate propagation of stressed cells. Because of this protein's vital role in maintaining normal cellular function, tumor cells have developed numerous methods to disable its function. Indeed, the p53 protein is inactivated by mutations or deletions in approximately 50% of human cancers [4]. A polymorphism at codon 72 with a single-base change in the *p53 *gene causes an amino acid replacement in the transaction domain of the protein Arg (CGC) with Pro (CCC). Although the functional differences of these two variants of the p53 protein remain unclear, it has been demonstrated that a single nucleotide polymorphism (SNP) at codon 72 of the *p53 *gene is associated with the risk for development of various neoplasms. However, in the rest of human tumor types, the *p53 *gene remains in a wild-type form and its activity is eradicated by its principal cellular inhibitor, murine double minute 2 protein (MDM2) [5].

MDM2 is the primary regulator of p53. MDM2 and p53 regulate each other through a feedback loop. In this mechanism, p53 induces MDM2, and MDM2 then acts as an E3 ubiquitin ligase that exports p53 out of the nucleus and promotes its degradation [6]. Moreover, MDM2 is capable of affecting genome stability in a p53-independent way [7]. A functional single-nucleotide T to G polymorphism is present in the promoter of the *MDM2 *gene (rs2279744), known as *MDM2 *SNP309[8]. Bond et al. demonstrated that the GG genotype of SNP309 enhanced the affinity of the transcription factor Sp1 to the *MDM2 *promoter in cell lines, and consequently enhanced the expression of MDM2 RNA and protein, resulting in a possible attenuation of the p53 pathway [9]. In both patients with hereditary Li-Fraumeni syndrome (one *p53 *allele mutated) and patients with sporadic soft tissue sarcoma, the presence of the SNP309 G-allele accelerated tumor formation [10,11].

To date, a number of studies have explored the association between *MDM2 *SNP309 and the risk of various types of cancer [12-77], including brain, breast, colorectal, hepatocellular, lung, ovarian, gastric, uterus, and so on. Nearly three years since the meta-analysis was performed by Hu et al. [78], forty-one additional case-control studies regarding the association between SNP309 and tumor risk have appeared, which is a greater number of studies than the number of studies included in the original meta-analysis. Therefore, an updated meta-analysis is needed. The meta-analysis presented in this study aims to assess whether *MDM2 *SNP309 is associated with cancer risk and to investigate the possible interaction between *MDM2 *SNP309 and *p53 *mutation status and the *p53 *codon72 polymorphism.

## Methods

### Primary search strategy

We searched the Pub Med and CNKI databases for all genetic association studies published to date on the *MDM2 *SNP309 and tumor risk (the most recent search update was April 2, 2010). To perform the search we used the subject terms "*MDM2 *polymorphism(s) and tumor". Only English-language and Chinese-language papers were included. The references cited in the original studies or review articles concerning the relevant topic were retrieved in order to potentially broaden the search with additional relevant publications.

### Criteria for study inclusion and exclusion

All studies reporting human associations that met the following criteria, regardless of sample size, were included in this meta analysis; if not, the studies were excluded: (a) the study is a case-control study on the association of *MDM2 *SNP309 and tumor susceptibility; (b) the study reports genotypic frequencies of *MDM2 *SNP309 in cancer patients and controls; (c) the genotype of the control population is in Hardy-Weinberg equilibrium. If the study had the same population resource or had overlapping subjects, only the study reporting the largest population was selected. Hence, we included sixty-six studies in our meta-analysis, containing 27,813 cases with different tumor types and 30,295 controls.

### Data extraction

Two reviewers independently extracted data using a standardized extraction form. For each case, if a disparity was identified the two reviewers debated until a consensus was reached on all items. The following information was collected from each publication: the first author's name, year of publication, tumor type, ethnicity, genotype frequency for cases and controls, minor allele frequency (MAF) in controls, *p53 *mutation status, and interaction with *p53 *Arg72Pro status (Additional file 1). If a study contained more than one tumor type or ethnicity, genotype data were extracted separately according to tumor type or ethnicity for subgroup analyses. Racial descent was classified as European, Asian, African, and mixed.

### Statistical analysis

Odds ratios (ORs) were pooled to evaluate the association between *MDM2 *SNP309 and tumor risk. The fixed effect model and the random effect model based on the Mantel-Haenszel method and the Dersimonian and Laird method, respectively, were used to pool data from different studies. If the heterogeneity between studies is absent, these two models provide similar results; otherwise, it is more appropriate to adopt the random effect model. We first compared the tumor risk in the variant homozygote GG and in the heterozygote TG with the wild-type TT homozygote. The ORs and 95% CIs were calculated. The statistical significance of the OR was determined using the Z test. Statistical heterogeneity between studies was assessed with the χ^2^-based Q test and Ι^2^, heterogeneity was considered significant when P < 0.1, and Ι^2 ^was used to qualify variation in OR attributable to heterogeneity. Crossover analysis was used for interaction analysis.

Publication bias was investigated using the funnel plot, a method used to analyze subjective data. To supplement the funnel plot method, we also adopted the liner regression approach proposed by Egger et al. ORs and 95%CIs were generated by meta-analysis using STATA (version 10.0).

## Results

### Characteristics of studies

**Figure 1:**
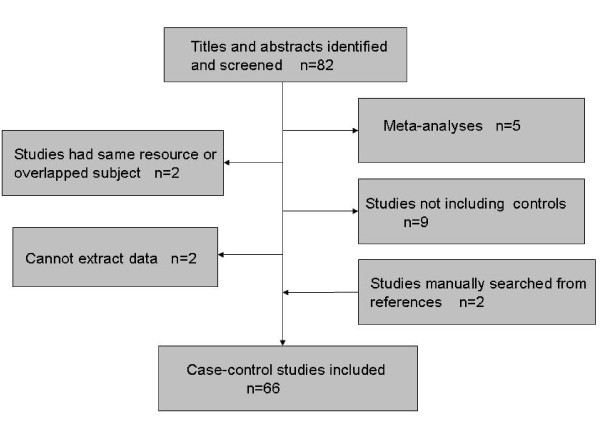
**Flow chart of the eligible study selection process**.

After screening the titles and abstracts, a total of eighty-two full text articles were reviewed to identify eligibility for our systematic review. According to the inclusion criteria, we found that five articles were meta-analyses [78-82], two studies utilized the same population resource or contained overlapping subjects, nine articles did not include controls, and for two studies we were unable to extract the data. In addition, we included two studies [23,35] that were retrieved manually from the original articles' references. Thus, for our systematic-review, we summarized the results of sixty-six case-control studies [12-77], including sixty-three English language articles and three Chinese language articles [36,39,57] and containing seventy-eight comparisons (Figure 1).

The detailed characteristics of the sixty-six case-control studies are shown in Additional file 1. These studies contain thirty tumor types, and the racial descent of the subjects is classified as European, Asian, African, and mixed. The genotype distribution observed in the controls was consistent with the Hardy-Weinberg equilibrium for all studies. There were ten studies in which the *p53 *mutation status was detected in tumor cases, but only seven of these studies presented the *MDM2 *SNP309 genotype distributions according to *p53 *mutation status [16,20,35,37,41,54,70]. There were twelve studies that investigated the interaction of *MDM2 *SNP309 and *p53 *codon72 polymorphism on cancer risk; however, only six studies [19,28,46,51,60,72] offered detailed data (Table 1).

**Table 1:** Datas for the interaction of *p53 *codon72 polymorphism and *MDM2 *SNP309 for tumor susceptibility

First author	Tumor site	309TT		309TG		309GG	
		
(reference )		Case*	Control*	Case*	Control*	Case*	Control*
Yang M	GCA	19/61/27	96/150/52	59/119/72	162/222/114	45/65/33	58/114/32
Singh V	BC	10/11/4	4/18/3	23/21/4	13/32/2	13/13/5	11/15/7
Yoon YJ	HCC	23/18/4	40/38/6	48/58/19	56/53/23	39/35/43	28/45/8
Xiong XJ	AML	6/22/4	12/17/6	29/62/32	22/33/13	17/43/16	5/14/6
Zhang X	LC	62/127/60	122/222/74	170/259/132	223/343/145	89/120/87	80/166/45
Cox DG	BC	349/218/40	488/346/60	317/266/45	539/365/52	104/63/14	166/92/10

*P53 codon72 wild-type homozygote/heterozygote/variant homozygote

### Quantitative synthesis

There was wide variation in the *MDM2 *309G allele frequency in the different ethnic groups (Additional file 1). The mean frequency of the G allele was 0.11 for African, 0.37 for European, 0.50 for Asian, and 0.36 for mixed ethnicities.

When all of the eligible studies were pooled, we found that the variant genotypes were associated with increased tumor risk in several genetic models. The variant homozygote GG exhibited a significantly increased risk for all tumor types when compared with the wild-type TT homozygote (OR = 1.25, 95%CI = 1.13-1.37; P < 0.001 for heterogeneity test; Ι^2 ^= 66.9% for heterogeneity). Interestingly, we also found that the variant heterozygote TG exhibited an increased risk for all tumor types (OR = 1.10, 95%CI = 1.03-1.17; P < 0.001 for heterogeneity test; Ι^2 ^= 51.7% for heterogeneity). Significant effects were also found both in the recessive and dominant models (recessive model: OR = 1.18, 95%CI = 1.10-1.27; P < 0.001 for heterogeneity test; Ι^2 ^= 55.1% for heterogeneity; dominant models: OR = 1.14, 95%CI = 1.07-1.22; P < 0.001 for heterogeneity test; Ι^2 ^= 62.2% for heterogeneity; Table 2).

**Table 2:** Summary OR (95%CI) and I-squre for various contrasts of the *MDM2 *SNP309 polymorphism and tumor risk

Subgroup	No. comparisons	TG vs. TT		GG vs. TT		GG vs. TT/TG		GG/TG vs. TT	
		
		OR (95% CI)	I-squre (%)	OR (95% CI)	I-squre (%)	OR (95% CI)	I-squre (%)	OR (95% CI)	I-squre (%)
**Total**	**78**	1.10* (1.03-1.17)	**51.7**	1.25* (1.13-1.37)	**66.9**	1.18* (1.10-1.27)	**55.1**	1.14* (1.07-1.22)	**62.2**
**Tumor site**									
**Breast**	**18**	1.09* (1.00-1.19)	**12.8**	**1.09 (0.96-1.24)**	**20.7**	**1.05 (0.94-1.17)**	**19.5**	1.09* (1.01-1.17)	**9.1**
**Lung**	**10**	**1.06 (0.93-1.22)**	**64.8**	**1.21 (0.99-1.47)**	**67.8**	**1.12 (0.99-1.27)**	**42.7**	**1.10 (0.95-1.27)**	**72.4**
**Ovarian**	**3**	**0.85 (0.63-1.16)**	**25.2**	**0.79 (0.48-1.28)**	**52.3**	**0.89 (0.63-1.25)**	**33.1**	**0.82 (0.57-1.18)**	**50.5**
**Pancreatic**	**2**	**1.55 (0.98-2.46)**	**59**	**1.56 (0.66-3.68)**	**74.6**	**1.24 (0.68-2.24)**	**56.4**	**1.54 (0.90-2.65)**	**73**
**Blood**	**4**	**1.08 (0.65-1.79)**	**79.4**	**1.11 (0.57-2.17)**	**80.3**	**1.09 (0.71-1.68)**	**63.8**	**1.10 (0.67-1.82)**	**81.6**
**Brain**	**5**	1.47* (1.06-2.03)	**57.6**	1.18* (1.08-3.03)	**69.5**	**1.38 (0.94-2.01)**	**55.9**	1.51* (1.10-2.07)	**61.3**
**Colorectal**	**6**	**1.14 (0.89-1.47)**	**51.2**	**1.05 (0.65-1.68)**	**74.4**	**1.00 (0.67-1.49)**	**70**	**1.13 (0.85-1.50)**	**65.5**
**Esophageal**	**2**	**1.11 (0.86-1.43)**	**36**	**1.26 (0.92-1.72)**	**51.2**	**1.28 (0.97-1.69)**	**63.1**	**1.18 (1.00-1.41)**	**0**
**Head-neck**	**4**	**0.92 (0.67-1.28)**	**71.6**	**1.01 (0.69-1.47)**	**72.3**	**1.11 (0.96-1.29)**	**1.7**	**0.94 (0.67-1.34)**	**77.8**
**Liver**	**4**	1.57* (1.18-2.09)	**0**	2.24*(1.57-3.18)	**0**	1.65* (1.25-2.17)	**0**	1.76* (1.34-2.31)	**0**
**Skin**	**4**	**1.00 (0.86-1.16)**	**0.4**	**1.05 (0.85-1.29)**	**1.2**	**1.05 (0.86-1.27)**	**0**	**1.01 (0.86-1.19)**	**26.6**
**Stomach**	**5**	**1.03 (0.75-1.42)**	**72.2**	1.54* (1.04-2.29)	**76.4**	1.49* (1.20-1.84)	**53.2**	**1.18 (0.84-1.65)**	**77.6**
**Uterus**	**8**	**0.95 (0.81-1.11)**	**0**	1.34* (1.07-1.69)	**5.3**	**1.26 (0.92-1.72)**	**51.2**	0.81* (0.70-0.94)	**0**
**Other**	**3**	**0.98 (0.72-1.32)**	**14.6**	**1.14 (0.63-2.08)**	**66.8**	**1.16 (0.72-1.85)**	**62.6**	**1.02 (0.67-1.52)**	**55**
**Racial descent**									
**African**	**3**	**1.22 (0.80-1.86)**	**61.2**	**0.75 (0.40-1.41)**	**0**	**0.73 (0.39-1.36)**	**0**	**1.16 (0.80-1.70)**	**56**
**European**	**34**	**1.05 (0.98-1.13)**	**34.8**	1.13* (1.01-1.25)	**40.8**	1.10* (1.00-1.20)	**30.8**	1.08* (1.00-1.16)	**42.1**
**Asian**	**32**	**1.11 (0.99-1.24)**	**58.4**	1.36* (1.18-1.56)	**68.6**	1.27* (1.15-1.40)	**58.6**	1.18* (1.05-1.32)	**65.8**
**Mixed**	**9**	**1.10 (0.91-1.32)**	**49.7**	**1.16 (0.86-1.56)**	**59.6**	**1.08 (0.88-1.35)**	**37.3**	**1.10 (0.92-1.32)**	**52.7**
**p53 mutation status**									
**Positive**	**7**	**1.09 (0.89-1.34)**	**0**	**1.17 (0.75-1.82)**	**59.4**	**1.09 (0.70-1.69)**	**70.3**	**1.12 (0.92-1.36)**	**0**
**Negative**	**7**	**1.06 (0.85-1.30)**	**4.1**	**0.95 (0.72-1.25)**	**0**	**0.97 (0.76-1.22)**	**0**	**1.03 (0.85-1.25)**	**0**

*P value < 0.05 for significance tests of OR = 1

‡ P value = 0.056 for significance test of OR = 1

Subsequently, we investigated the effects of *MDM2 *SNP309 stratified by tumor location, ethnicity, and *p53 *mutation status. We found that there was an association between individuals with the GG genotype or TG genotype and an elevated risk of breast, brain, liver, stomach, and uterus cancer when compared to subjects with the TT genotype (Table 2). Interestingly, the risk was significant in brain, liver, and stomach cancer. Regarding the different ethnic groups, we found a subtle cancer risk in the European population (OR = 1.13, 95%CI = 1.01-1.25) and a significant cancer risk in the Asian population (OR = 1.36, 95%CI = 1.18-1.56). However, no significant associations were found in either the *p53 *mutation-positive or *p53 *mutation-negative subgroup (Table 2).

### Gene-gene interaction

In this meta-analysis, we pooled the eligible studies for association of *MDM2 *SNP309 and *p53 *Arg72Pro on tumor risk. In comparison to the reference *MDM2 *309TT and *p53 *Arg/Arg genotype, the OR (3.38) for subjects with the *MDM2 *309GG and p53 Pro/Pro genotype is larger than the OR (1.96) for subjects with the *MDM2 *309GG and p53 Arg/Arg or the OR (1.38) for subjects with the *MDM2 *309TT and p53 Pro/Pro genotype (Table 3). These results indicate a possible compounding effect between the *MDM2 *309GG and *p53 *Pro/Pro genotype that leads to a significantly increased risk of cancer. The p-value for the overall interaction analysis is less than 0.001.

**Table 3:** Interaction of *MDM2 *SNP309 (T to G) and *p53 *Arg72Pro on tumor risk

*MDM2 *309T > G	*P53 *72Arg > Pro	Case	Control	OR (95% CI)
TT	Arg/Arg	469	762	Reference
TT	Arg/Pro	457	791	1.09 (0.74-1.61)
TT	Pro/Pro	139	201	1.38 (0.92-2.06)
TG	Arg/Arg	646	1015	1.32 (0.88-1.98)
TG	Arg/Pro	785	1048	1.51 (0.96-2.38)
TG	Pro/Pro	304	349	1.88 (1.26-2.81)*
GG	Arg/Arg	307	348	1.96 (1.01-3.78)*
GG	Arg/Pro	339	446	1.53 (0.92-2.53)
GG	Pro/Pro	198	108	3.38 (1.77-6.47)*

*P values < 0.05 for significance test of OR = 1

### Test of heterogeneity

We observed heterogeneity between studies regarding both overall comparisons and subgroup analyses. Hence, the random effect model based on the Mantel-Haenszel method was adopted for this meta-analysis. The details of Ι^2 ^for each comparison are shown in Table 2.

### Publication bias

**Figure 2:**
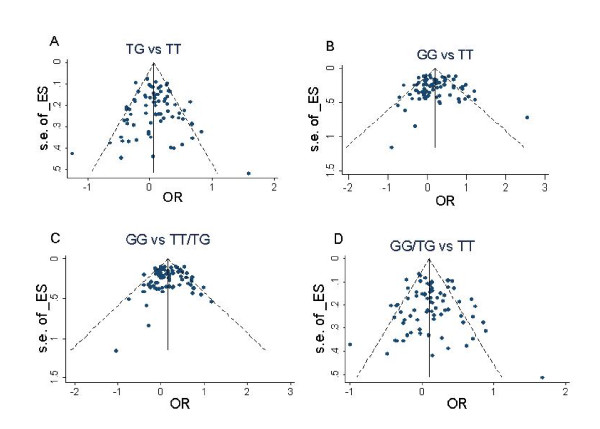
**Funnel plot of association between MDM2 SNP309 and cancer risk**.

The publication bias of the studies was determined by the Funnel plot and Egger's test. As shown in Figure 2 (A/B/C/D), the shapes of the funnel plots appeared symmetrical in all comparisons, indicating the absence of publication bias. Next, we used Egger's test to provide statistical evidence for the funnel plot symmetry. The greater the intercept deviation from zero in linear regression analysis, the greater the possibility for asymmetry. We considered the funnel plot to be symmetrical if we observed a 95% confidence interval with an intercept of zero. The results are shown in Table 4.

**Table 4:** The results of egger's test for four comparisons

Comparison type	Intercept value	t-value	P-value	95% CI of intercept value
TG vs. TT	0.24	0.67	0.507	-0.49 ~ 0.98
GG vs. TT	-0.07	-0.16	0.87	-0.91 ~ 0.77
GG vs. TT/TG	-0.22	-0.6	0.55	-0.94 ~ 0.51
GG/TG vs. TT	0.35	0.87	0.39	-0.45 ~ 1.15

## Discussion

On the basis of sixty-six case-control studies focused on *MDM2 *309 T/G polymorphism and tumor risk, our meta-analysis provided evidence that the variant homozygote GG and heterozygote TG were significantly associated with increased tumor risk. Our findings are in concordance with the meta-analysis conducted by Hu et al. which suggests that the *MDM2 *SNP309 serves as a low-penetrance susceptibility tumor marker [78].

When stratified according to ethnicity, our meta-analysis showed that the GG genotype was significantly associated with tumor risk in the Asian and European populations. According to some previously reported studies, the GG genotype is significantly associated with tumor risk in the Asian population, but not in the European population [19,80,82]. One possible reason is that our meta-analysis includes thirty-four European study comparisons, a large number of studies in comparison to previously reported comparisons. Consistent with previously reported studies, no significant associations were found in the African ethnic group [78]. This may suggest a potential role for ethnic differences in genetic backgrounds as well as environmental exposures. The mean MAF in the African group was 0.10, whereas in the Asian and European groups it was 0.50 and 0.38, respectively. The large differences in the MAF between the African group and the Asian or European group may be a result of natural selection pressures, or balance due to other related genetic variants. Therefore, further studies regarding the *MDM2 *309T/G polymorphism in Africans and the underlying mechanism for ethnic differences are warranted.

In our meta-analysis, we included approximately thirty tumor types stratified into fourteen subgroups according to tumor location. In the subgroup analyses, we found a significant association in breast cancer but a non-significant association in lung cancer and colorectal cancer. Interestingly, our meta-analysis determined a significant association in brain, liver, stomach, and uterus cancer. The ORs for the GG genotype compared to TT was 1.18, 2.24, 1.54, and 1.34, respectively. Similar findings have been reported in previous studies, including hepatocellular carcinoma associated with chronic hepatitis C, gastric carcinoma, and sporadic endometrial carcinoma [49,70,75], suggesting an interaction of *MDM2 *SNP309, infectious factor, and hormone factor.

In order to incorporate the p53 mutation status while investigating the effect of *MDM2 *SNP309 on tumors, we included seven studies to pool the patient genotypes according to the *p53 *mutations [16,20,21,35,37,41,54,70]. However, we found no discrepancy between the two *p53 *mutation groups, possibly due to insufficient statistical power. Furthermore, the functional polymorphism of the *p53 *codon 72 (Arg72Pro) had been shown to interact with SNP309 in the carcinogenesis of several carcinomas [46,51,60,72]. Our meta-analysis included six studies that explored interaction effects between *p53 *Arg72Pro and *MDM2 *SNP309. We found that the OR for subjects with the *MDM2 *309 GG genotype and *p53 *72 Pro/Pro genotypes compared to subjects with *MDM2 *309 TT and *p53 *72Arg/Arg genotypes (3.38) was larger than the OR for subjects with the *MDM2 *309GG genotype and *p53 *72Arg/Arg (1.96) or the OR for those with *MDM2 *309TT and *p53 *72 Pro/Pro (1.38). These results suggested a possible interaction effect between the *MDM2 *309GG and the *p53 *72 Pro/Pro genotype in increasing the risk of carcinogenesis.

The strength of our meta-analysis is due to the large number of comparisons included. However, our study does have a limitation: the controls in the studies included were not uniformly defined and thus the results presented here are based upon unadjusted estimates. A more precise analysis could be conducted with estimates adjusted according to covariates such as age, smoking, lifestyle, and environmental factors.

## Conclusions

In summary, our results provide some support for the hypothesis that *MDM2 *SNP309 is associated with tumor risk and support the potential interaction effect between the *MDM2 *SNP309 and the polymorphism of *p53 *codon72. This investigation could be extended in future studies by incorporating other potential risk factors and *p53*-*MDM2*-related genes for tumor development.

## Competing interests

We declare that we have no financial and personal relationships with other people or organizations that can inappropriately influence our work; there is no professional or other personal interest of any nature or kind in any product, service and/or company that could be construed as influencing the position presented in, or the review of the manuscript. The authors indicated no potential conflicts of interest.

## Authors' contributions

YW carries out the meta-analysis study and drafted the manuscript. WW participates in the design of the study and performs the statistical analysis. ZY and WW collect and extract the data. PG has been involved in revising the manuscript critically for important intellectual content. BZ conceives of the study, and participates in its design and coordination and helps to draft the manuscript. All authors read and approve the final manuscript.

## Pre-publication history

The pre-publication history for this paper can be accessed here:

http://www.biomedcentral.com/1471-2407/11/208/prepub

## Supplementary Material

Additional file 1 **Characteristics of included studies investigating the association between MDM2 SNP309 and tumor risk**. Click here for file
